# Supplementary material for: Supramolecular polynuclear clusters sustained cubic hydrogen bonded frameworks with octahedral cages for reversible photochromism
Source: Nat Commun. 2024 Mar 30;15:2782. doi: 10.1038/s41467-024-47058-1 (PMC10981757; doi:10.1038/s41467-024-47058-1)
Supplement: Supplementary file 1 — Supplementary Information [file 41467_2024_47058_MOESM1_ESM.pdf]

Supplementary Information

Supramolecular Polynuclear Clusters Sustained Cubic Hydrogen Bonded Frameworks with Octahedral Cages for Reversible Photochromism

Xiaojun Ding[1]*, Jing Chen[1], and Gang Ye[1]*

[1]Collaborative Innovation Center of Advanced Nuclear Energy Technology, Institute of Nuclear and New Energy Technology, Tsinghua University, Beijing 100084, China

Contents

Supplementary Table 1. Single crystal data of TCA_NH_4 3
Supplementary Figure 1. Images of prepared crystals........ 4
Supplementary Figure 2. Microscope picture of TCA_NH_4 4
Supplementary Table 2. Hydrogen bonds parameters in TCA_NH_4........ 5
Supplementary Figure 3. Enclosed octahedral cages........ 5
Supplementary Figure 4. Topological networks of TCA_NH_4 6
Supplementary Figure 5. SSBU structure of TCA_NH_4........ 6
Supplementary Figure 6. SSBU with interlaced orthogonally structure........ 7
Supplementary Table 3. C-H…N hydrogen bond parameters........ 7
Supplementary Figure 7. CO_2 adsorption isortherm of TCA_NH_4 8
Supplementary Figure 8. Truncated octahedral and encapsulated configuration........ 8
Supplementary Table 4. C-H…I hydrogen bond parameters........ 9
Supplementary Table 5. Crystal parameters of different samples........ 9
Supplementary Figure 9. Powder X-ray diffraction of different crystals........ 10
Supplementary Figure 10. Thermogravimetric analysis of different crystals........ 10
Supplementary Figure 11. PXRD of TCA_NH_4 after immersing in solvents........ 11
Supplementary Figure 12. The distances of adjacent halogen atoms........ 11
Supplementary Figure 13. Solid state fluorescence reflection spectra of different crystals........ 12
Supplementary Figure 14. Excitation spectra of different crystals........ 12
Supplementary Figure 15. Solid-state absorption spectra of different crystals........ 13
Supplementary Table 6. Lifetime fitting of different crystals........ 13
Supplementary Figure 16. EPR spectrum of TCA_NH_4@CHI_3........ 14
Supplementary Figure 17. EPR spectrum of TCA_NH_4@$CHCl_3$........ 15
Supplementary Figure 18. EPR spectrum of TCA_NH_4@$CHBr_3$........ 16
Supplementary Figure 19. PXRD of TCA_NH_4@CHI_3 before and after irradiation........ 17
Supplementary Figure 20. 5 times photochromic images of TCA_NH_4@CHI_3........ 17
Supplementary Table 7. Photosensitive efficiency of TCA_NH_4@CHI_3........ 18
Supplementary Figure 21. Solid state absorption spectroscopy of TCA_NH_4@CHI_3 after recovery........ 18
Supplementary Figure 22. Solid-state absorption spectrum of powder CHI_3........ 19
Supplementary Figure 23. The normalized emission spectrum of TCA_NH_4@CHI_3........ 19
Supplementary Figure 24. The excitation spectrum of TCA_NH_4@CHI_3........ 20

Supplementary Table 1. Crystallographic data and structure refinement data of TCA_NH_4.

**Single crystal**	**TCA_NH_4**
Empirical formula	$C_{84}H_{81}N_{10}O_{27}$
Formula weight	1722.67
Temperature/K	170.00
Crystal system	cubic
Space group	I23
a/Å	20.9737(2)
b/Å	20.9737(2)
c/Å	20.9737(2)
α/°	90
β/°	90
γ/°	90
Volume/$Å^3$	9226.2(3)
Z	4
ρ_{calc} g/cm^3	1.241
μ/mm^{-1}	0.787
F(000)	3616.0
Crystal size/mm^3	0.2 × 0.16 × 0.15
Radiation	CuKα (λ = 1.54178)
2Θ range for data collection/°	10.33 to 132.888
Index ranges	$-23 \leqslant h \leqslant 16, -22 \leqslant k \leqslant 24, -20 \leqslant l \leqslant 24$
Reflections collected	21578
Independent reflections	2724 [R_{int} = 0.1528, R_{sigma} = 0.0642]
Data/restraints/parameters	2724/0/184
Goodness-of-fit on F^2	1.020
Final R indexes [I>=2σ (I)]	R_1 = 0.0745, wR_2 = 0.2109
Final R indexes [all data]	R_1 = 0.0932, wR_2 = 0.2271
Largest diff. peak/hole / e $Å^{-3}$	0.24/-0.31
Flack parameter	0.2(2)
CCDC number	2309944

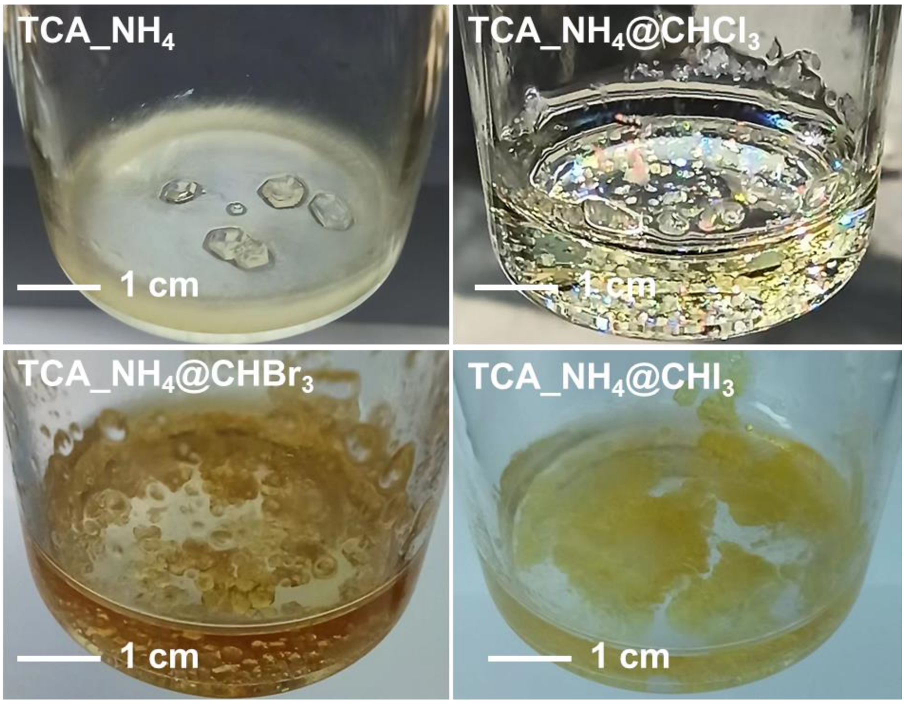

Supplementary Figure 1. Images of prepared TCA_NH_4, TCA_NH_4@$CHCl_3$, TCA_NH_4@$CHBr_3$, and TCA_NH_4@CHI_3.

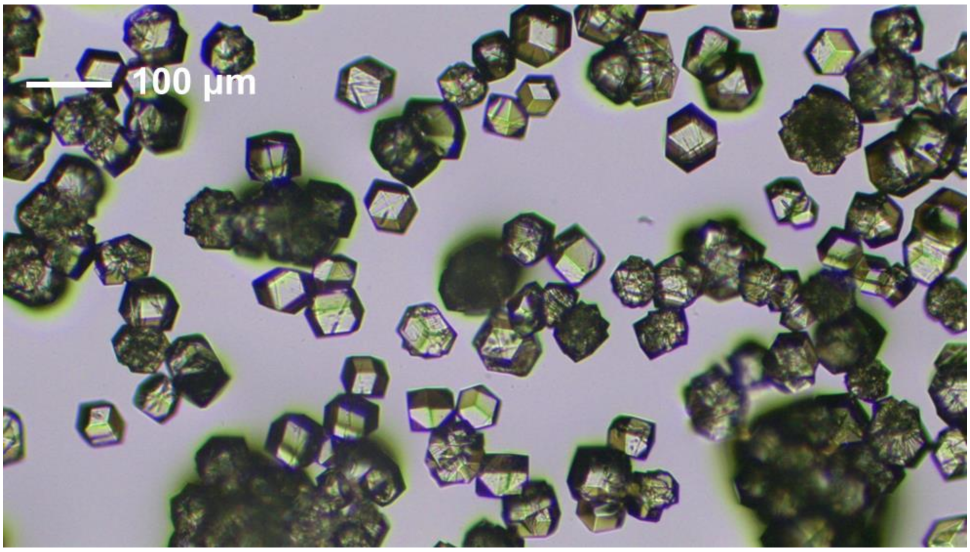

Supplementary Figure 2. A microscope picture of synthesized cubic TCA_NH_4.

Supplementary Table 2. Hydrogen bonds parameters in TCA_NH$_4$.

Donor	H	Acceptor	d(D-H)/ Å	d(H-A)/ Å	d(D-A)/ Å	D-H-A/°
N006[1]	H00C	O003	0.909	1.830	2.739	179.6
N006	H00F	O004[2]	0.911	2.509	3.323	148.9
N006	H00A	O00M[3]	0.910	2.477	3.235	140.8
O00M	H00M	O004[4]	0.718	2.069	2.756	160.9

[1]-z,1+x, -y; [2]1/2-x, 1/2-y, -1/2+z; [3]-1/2+x, 1/2-y, -1/2-z; [4]1/2-x, -1/2+y, -1/2-z

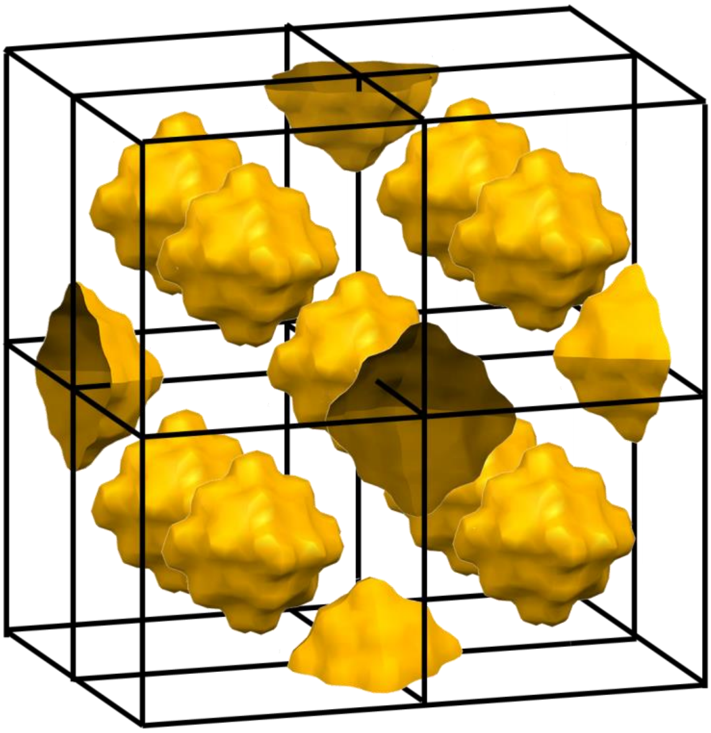

Supplementary Figure 3. Enclosed octahedral cages with size of about 12 Å in 2×2×2 lattice, depicted by mercury software with probe radius of 1.2 Å. All atoms were omitted for clarity.

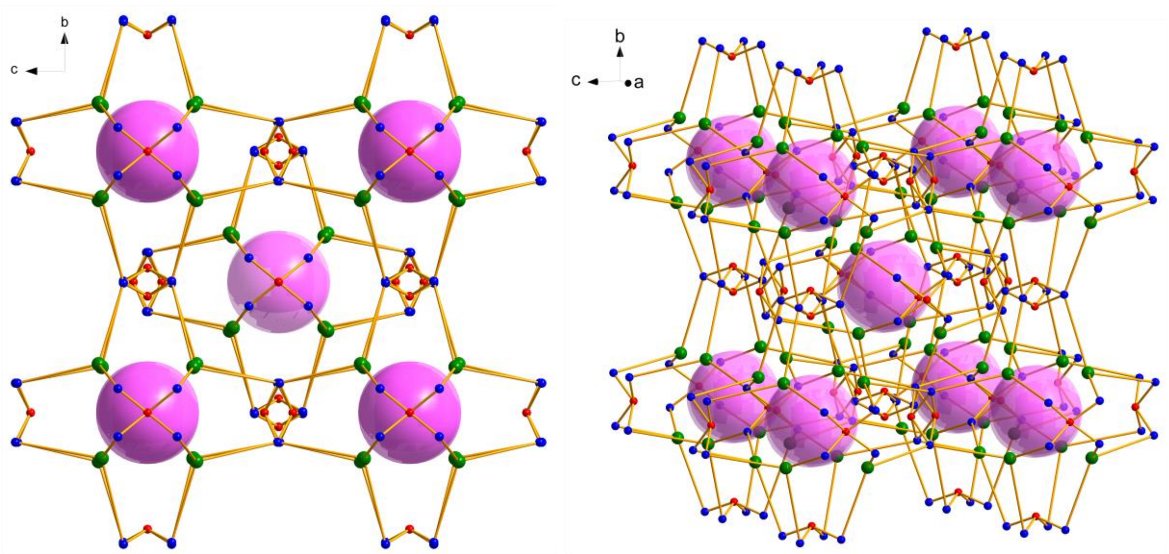

Supplementary Figure 4. Topological 2×2×2 networks of body-centered cubic TCA_NH_4 crystal lattices with simplified SSBU-NH_4-1 connecting adjacent cells along six directions of the plane. Nonhydrogen bonding hydrogen atoms are omitted for clarity.

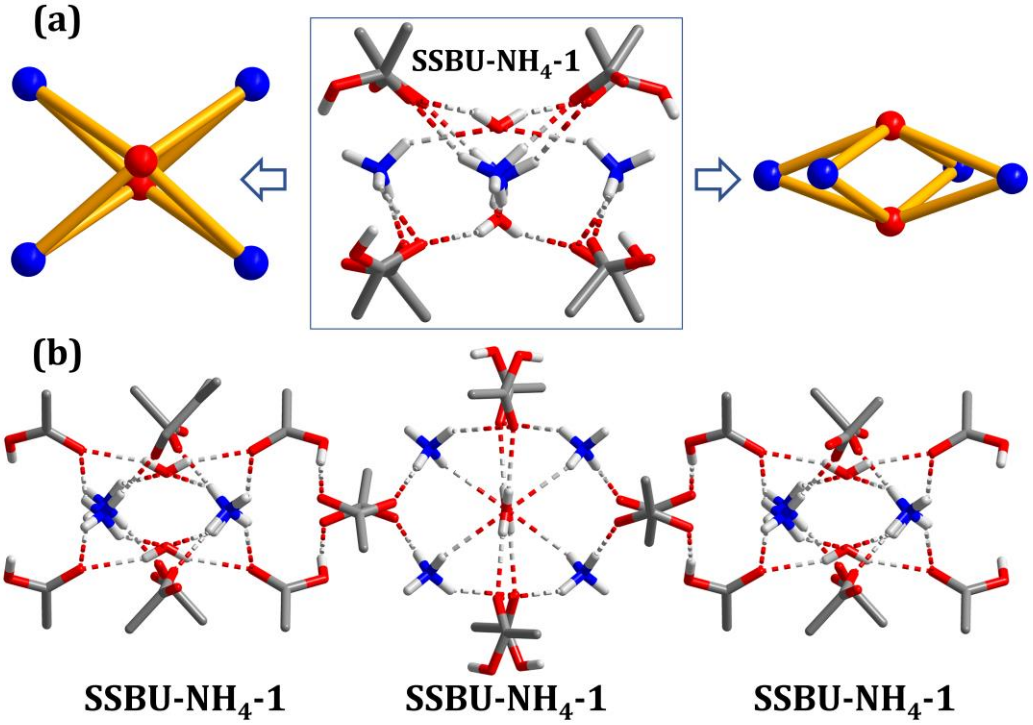

Supplementary Figure 5. (a) SSBU-NH_4-1 with concentrated polynuclear clusters and simplified two oxygen four nitrogen model. (b) Orthogonally connected SSBU-NH_4-1 through intermolecular carboxylic O-H…O hydrogen bonds as in Supplementary Figure 1b and **Supplementary Figure 4**.

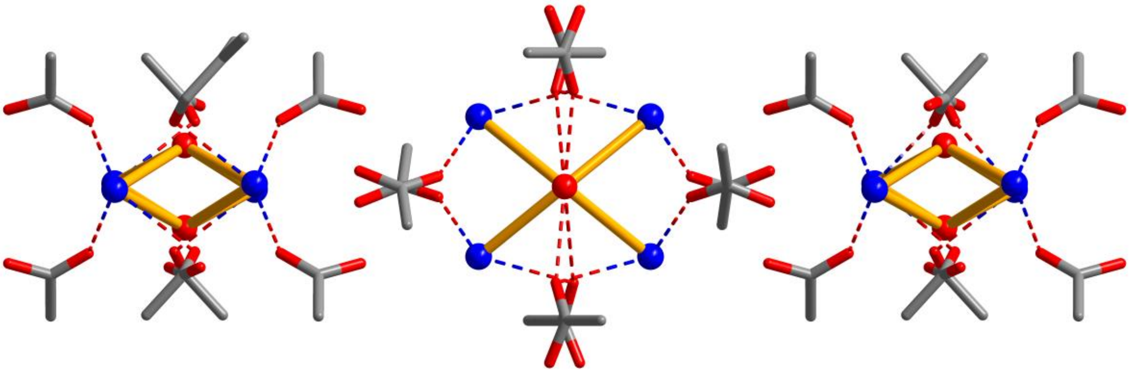

Supplementary Figure 6. Simplified SSBU-NH_4-1 interlaced orthogonally and O-H…O hydrogen bonds were omitted. All hydrogen atoms are also omitted for clarity.

Supplementary Table 3. C-H…N hydrogen bond parameters of TCA_NH_4@$CHCl_3$, TCA_NH_4@$CHBr_3$, and TCA_NH_4@CHI_3.

	d/C-H…N	θ/C-H…N	Unit Cell Volume
**TCA_NH_4**	--	--	9226.2 Å^3
**TCA_NH_4@$CHCl_3$**	2.805 Å	180°	9221.2 Å^3
**TCA_NH_4@$CHBr_3$**	3.007 Å	180°	9207.7 Å^3
**TCA_NH_4@CHI_3**	2.244 Å	180°	9204.7 Å^3

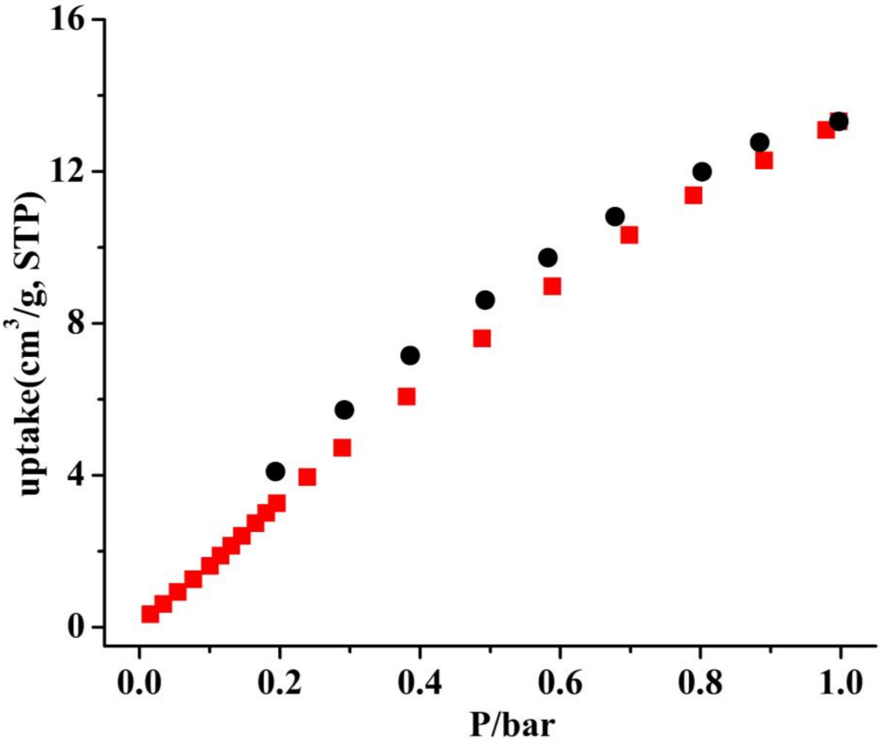

Supplementary Figure 7. CO_2 adsorption isortherm at 273 K of TCA_NH_4 (red adsorption, black desorption).

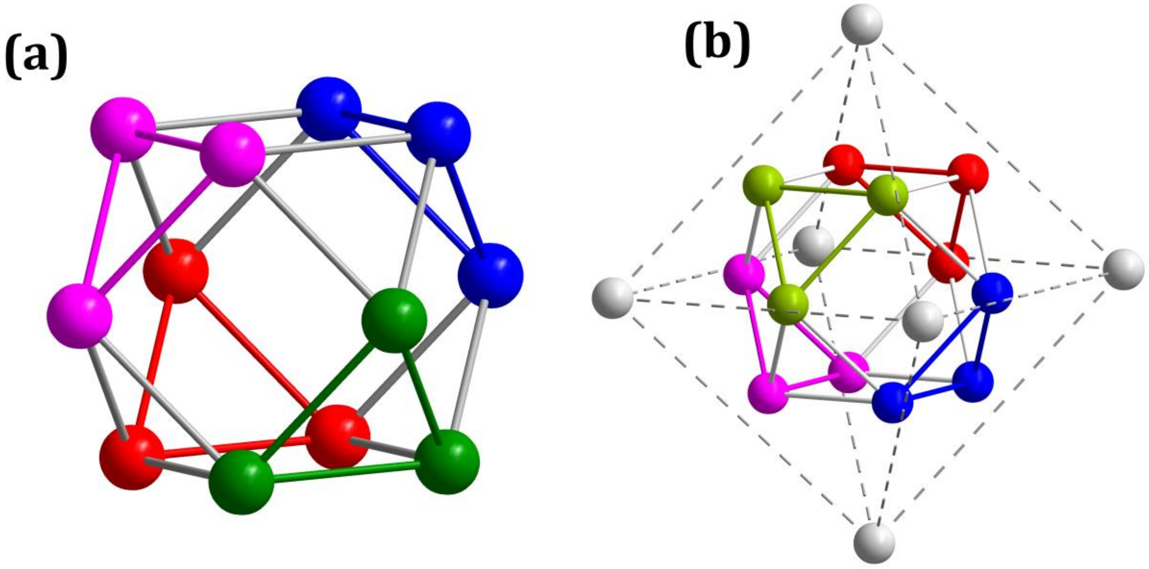

Supplementary Figure 8. (a) Truncated octahedral configuration and (b) encapsulated truncated octahedron within the cavity four CHX_3 (X=Cl, Br, I) in different colors.

Supplementary Table 4. C-H…I hydrogen bond parameters of $TCA\_NH_4@CHI_3$.

Donor	H	Acceptor	d(D-H)/ Å	d(H-A)/ Å	d(D-A)/ Å	D-H-A/°
C00K[1]	H00K	I1	0.930	2.817	3.600	142.4

[1]1+y, -z, 1-x;

Supplementary Table 5. Crystal parameters of TCA_NH_4, $TCA\_NH_4@CHCl_3$, $TCA\_NH_4@CHBr_3$, and $TCA\_NH_4@CHI_3$.

	**TCA_NH_4**	**$TCA\_NH_4@CHCl_3$**	**$TCA\_NH_4@CHBr_3$**	**$TCA\_NH_4@CHI_3$**
**Crystal system**	cubic	cubic	cubic	cubic
**Space group**	I23	I23	I23	I23
**a/Å**	20.9737(2)	20.9699 (4)	20.9596 (12)	20.9574 (1)
**b/Å**	20.9737(2)	20.9699 (4)	20.9596 (12)	20.9574 (1)
**c/Å**	20.9737(2)	20.9699 (4)	20.9596 (12)	20.9574 (1)
**α/°**	90	90	90	90
**β/°**	90	90	90	90
**γ/°**	90	90	90	90
**Volume/Å^3**	9226.2(3)	9221.2	9207.7	9204.7

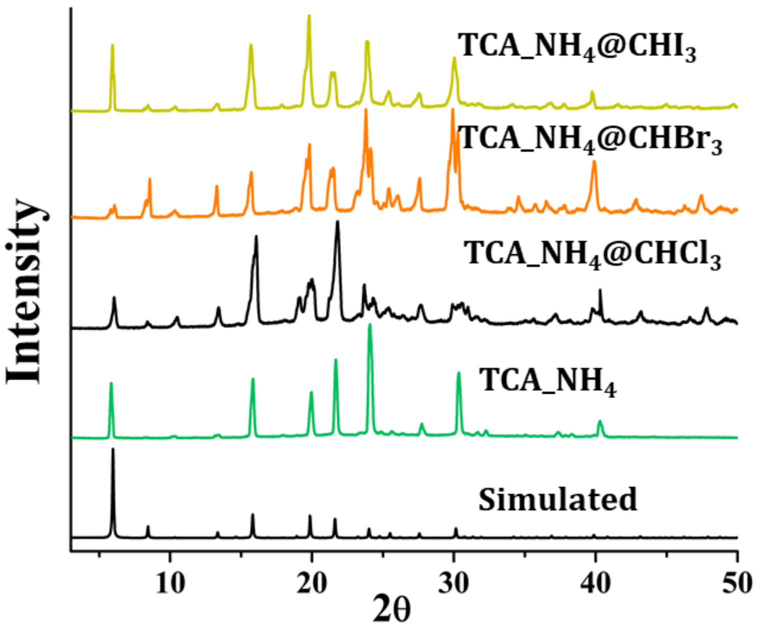

Supplementary Figure 9. Powder X-ray diffraction of simulated TCA_NH$_4$, measured TCA_NH$_4$, TCA_NH$_4$@CHCl$_3$, TCA_NH$_4$@CHBr$_3$, and TCA_NH$_4$@CHI$_3$.

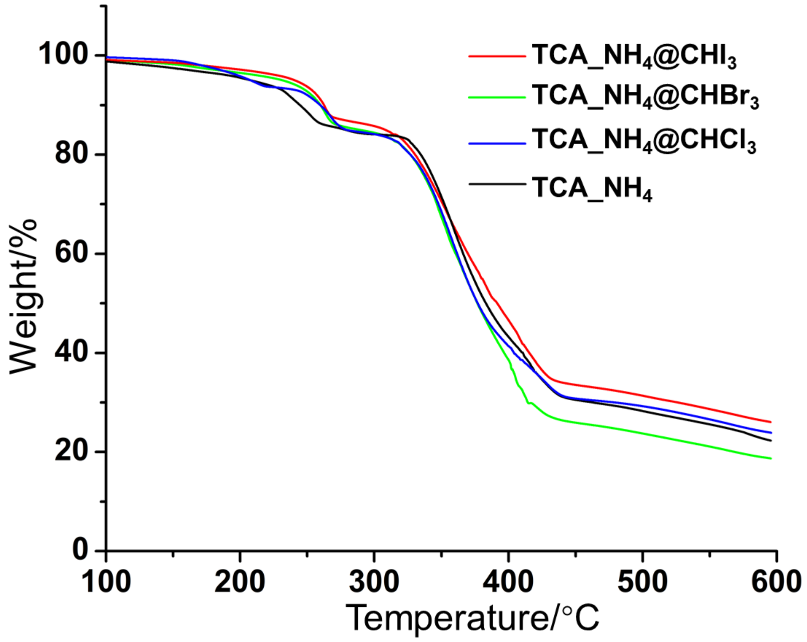

Supplementary Figure 10. Thermogravimetric analysis of TCA_NH$_4$, TCA_NH$_4$@CHCl$_3$, TCA_NH$_4$@CHBr$_3$, and TCA_NH$_4$@CHI$_3$ under N_2 gas atmosphere with flow of 10ml/min.

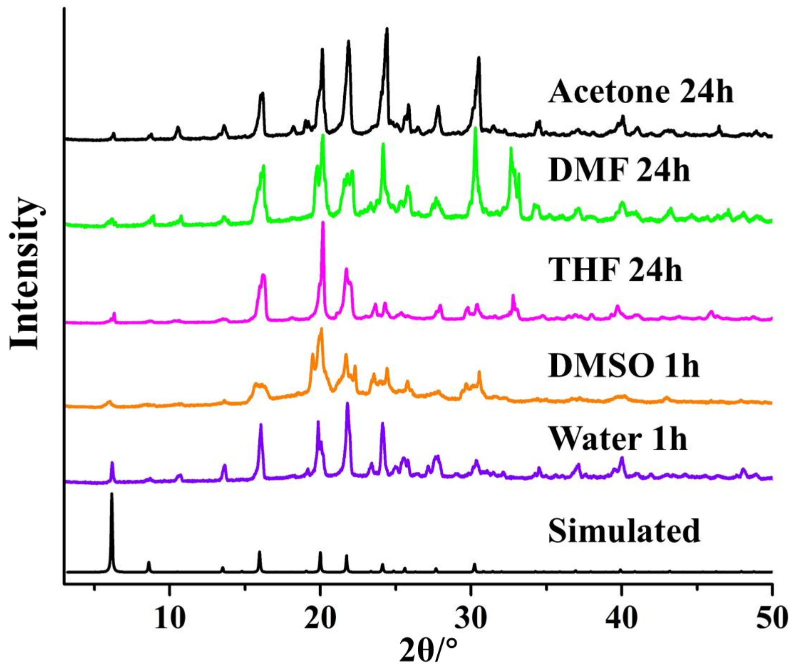

Supplementary Figure 11. PXRD of TCA_NH_4 simulated and after immersing in different solvent.

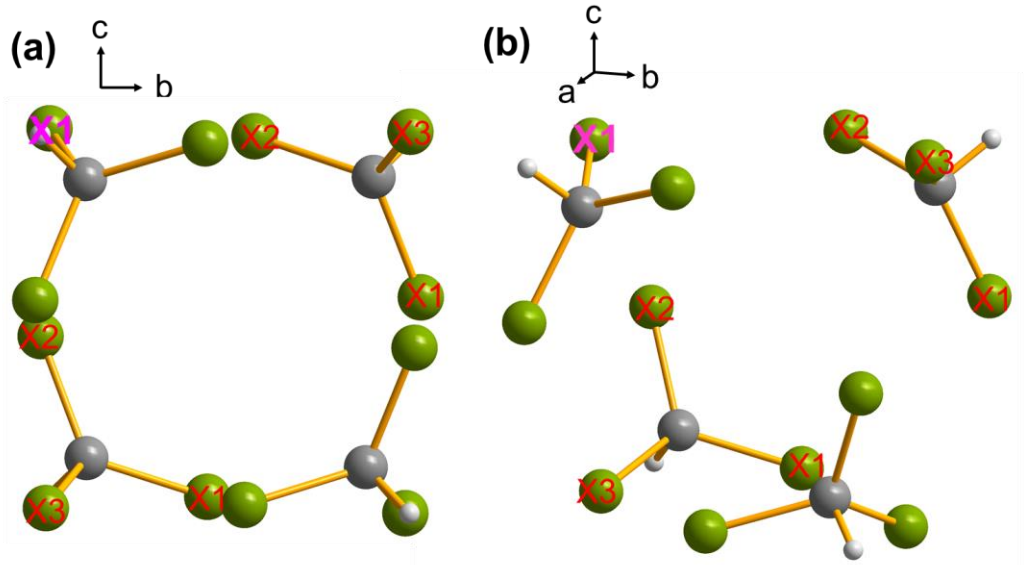

Supplementary Figure 12. The distances of adjacent halogen atoms with highlighted X1 (X= Cl, Br, I) as center viewed along ***a*** axis (a) and viewed along other direction (b).

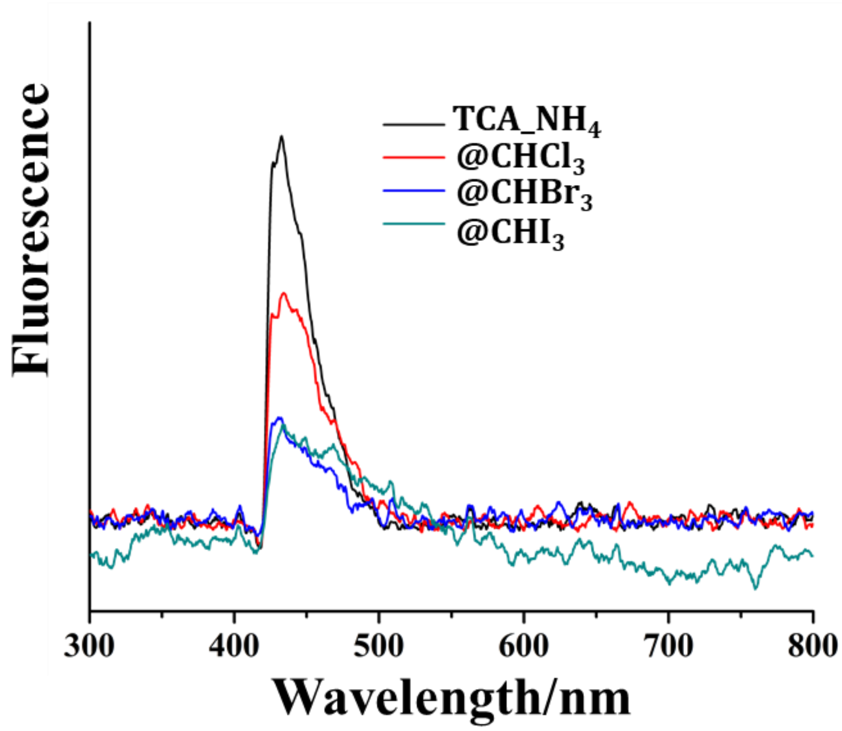

Supplementary Figure 13. Solid state fluorescence reflection spectra of TCA_NH_4, TCA_NH_4@$CHCl_3$, TCA_NH_4@$CHBr_3$, and TCA_NH_4@CHI_3.

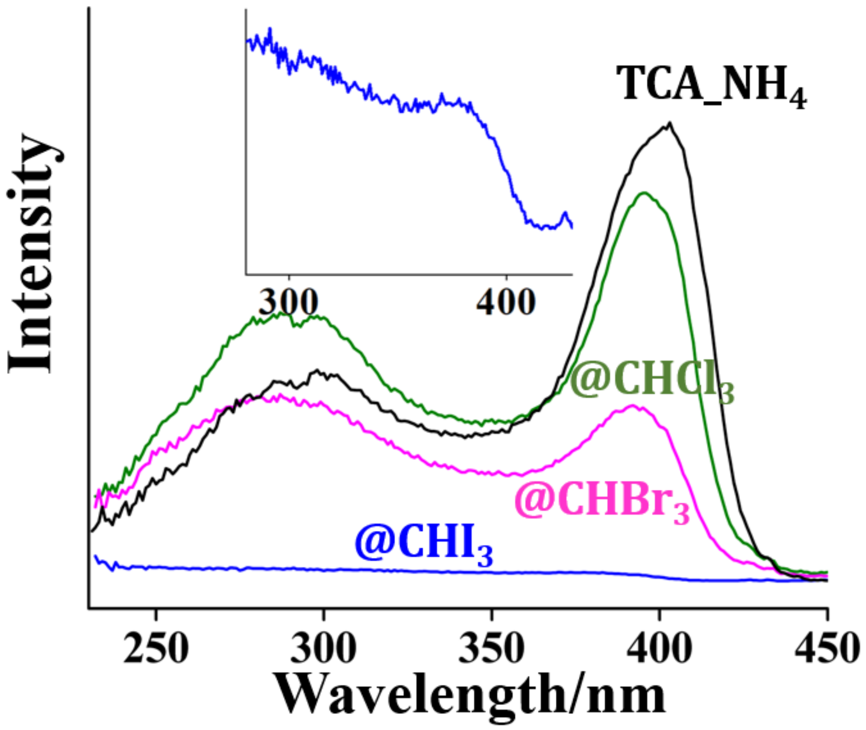

Supplementary Figure 14. Excitation spectra of TCA_NH_4, TCA_NH_4@$CHCl_3$, TCA_NH_4@$CHBr_3$, and TCA_NH_4@CHI_3 emission at 470 nm.

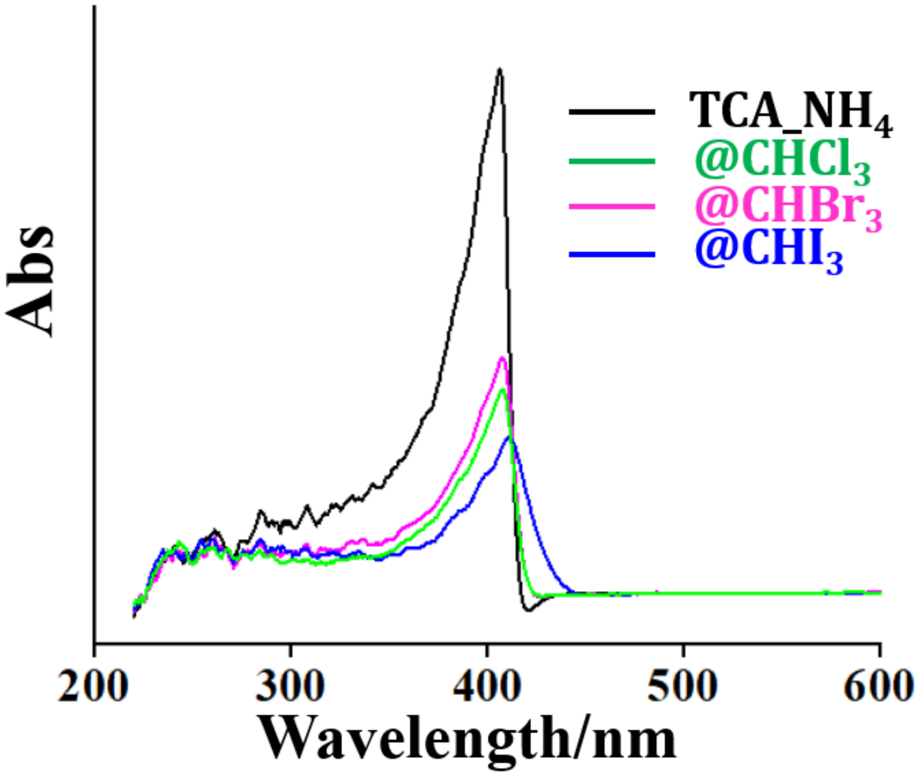

Supplementary Figure 15. Solid-state absorption spectra of TCA_NH_4, TCA_NH_4@$CHCl_3$, TCA_NH_4@$CHBr_3$, and TCA_NH_4@CHI_3.

Supplementary Table 6. Lifetime fitting of different crystals with time constants (τ), pre-exponential factor (B), and correlation coefficient (χ^2).

	τ/ns	B	A	χ^2
**TCA_NH_4**	2.092	2046	1.575	1.093
**TCA_NH_4@$CHCl_3$**	2.051	915.3	0.837	1.136
**TCA_NH_4@$CHBr_3$**	1.694	806.6	0.742	1.065
**TCA_NH_4@CHI_3**	2.433	998.9	0.845	1.069
**TCA_NH_4@I_2**	2.369	962.5	0.977	0.978

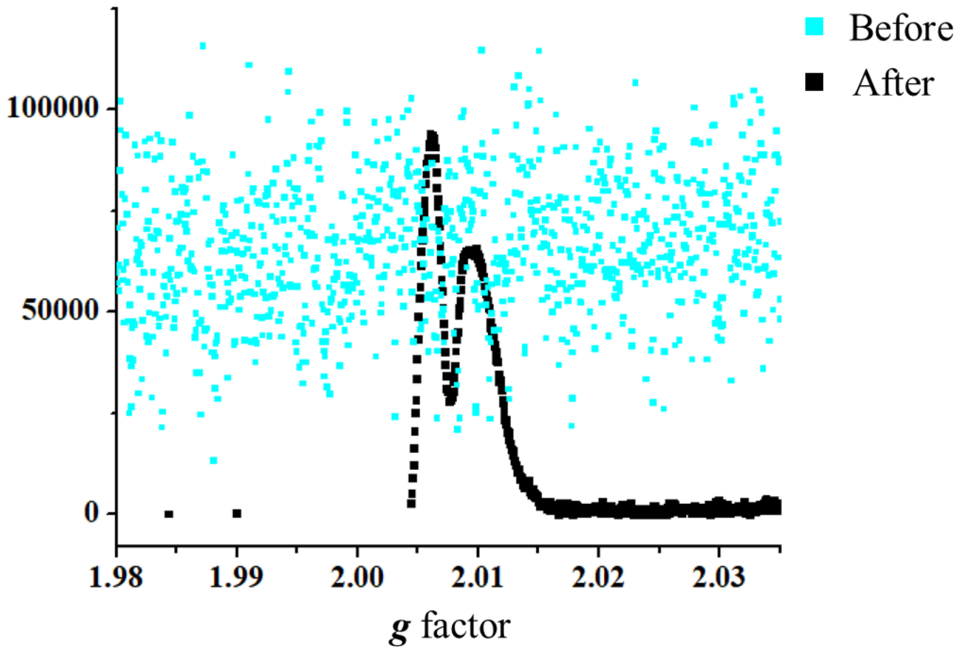

Supplementary Figure 16. EPR spectrum of TCA_NH_4@CHI_3 before and after irradiation with UV-vis lamp with power density of 200 mW/cm^2.

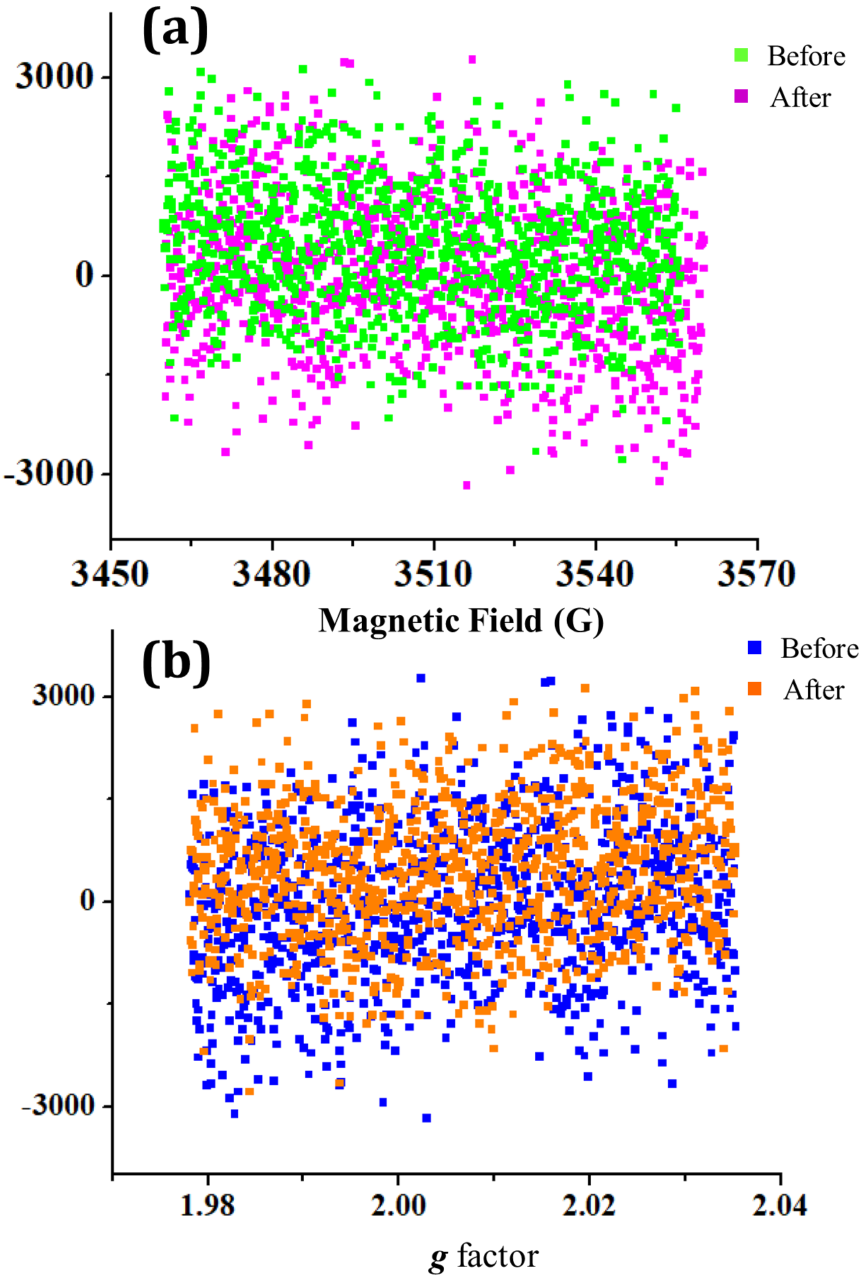

Supplementary Figure 17. EPR spectrum with magnetic field (a) and value ***g*** signals (b) of TCA_NH$_4$@CHCl$_3$ before and after irradiation with UV-vis lamp power density of 200 mW/cm^2.

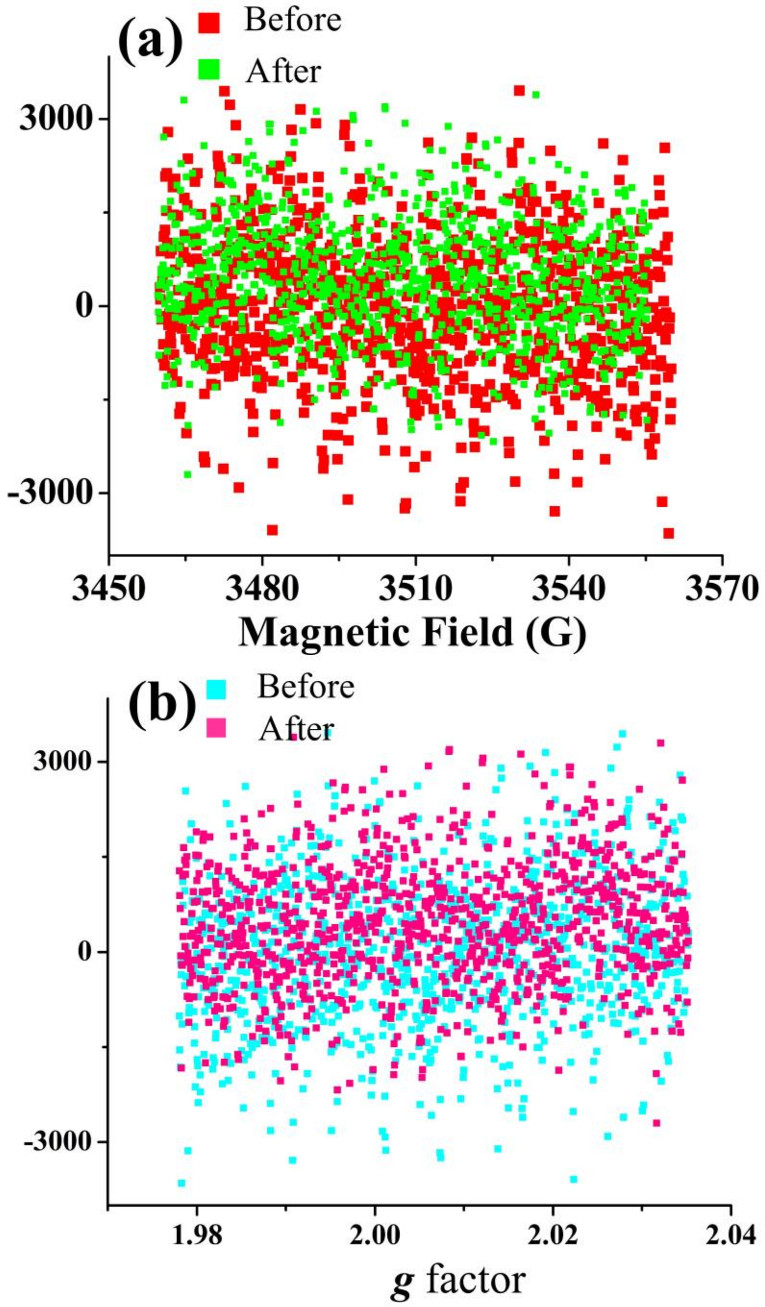

Supplementary Figure 18. EPR spectrum with magnetic field (a) and value ***g*** signals (b) of TCA_NH_4@$CHBr_3$ before and after irradiation with UV-vis lamp power density of 200 mW/cm^2.

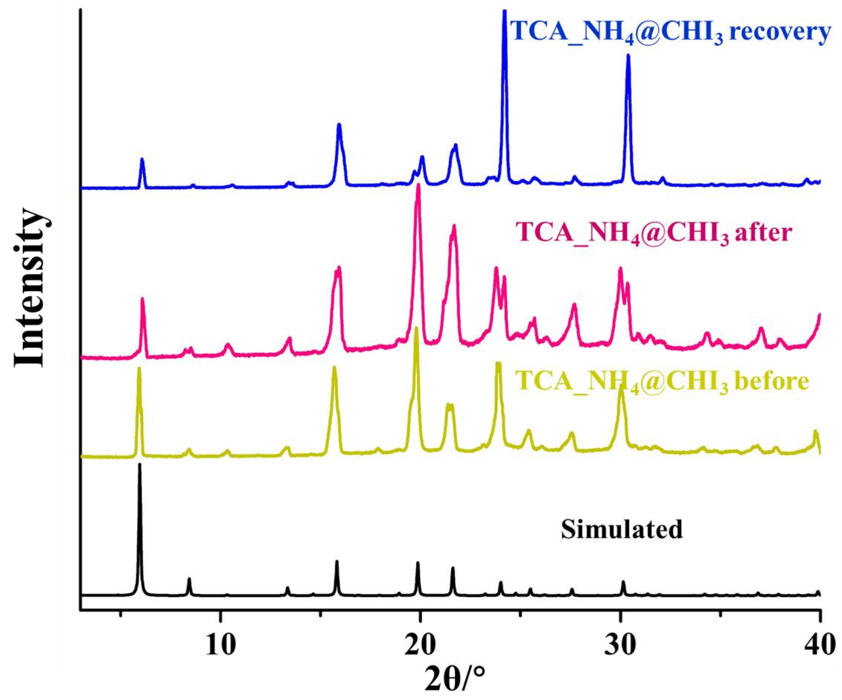

Supplementary Figure 19. PXRD of TCA_NH$_4$@CHI$_3$ before and after irradiation under Xe lamp and after thermal recovery.

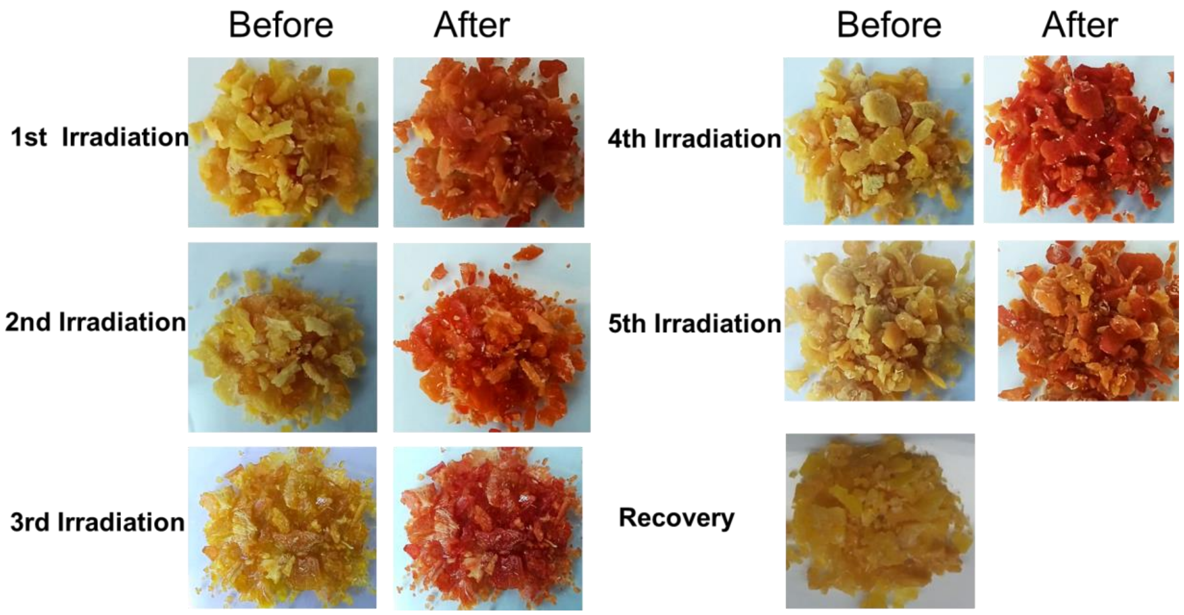

Supplementary Figure 20. 5 times photochromic images of TCA_NH$_4$@CHI$_3$ before irradiation and after irradiation under Xe lamp.

Supplementary Table 7. Photosensitive efficiency of TCA_NH_4@CHI_3 with different light sources.

Source	λ/nm	light power density/mW cm^2	Photochromic time
X ray	1.54 (CuKα)		30 mins
ultraviolet light	365	5	5 mins
sun light	290-2500	100	2 mins
LED	450-460	8	3 mins
Xe lamp	400	200	60 seconds

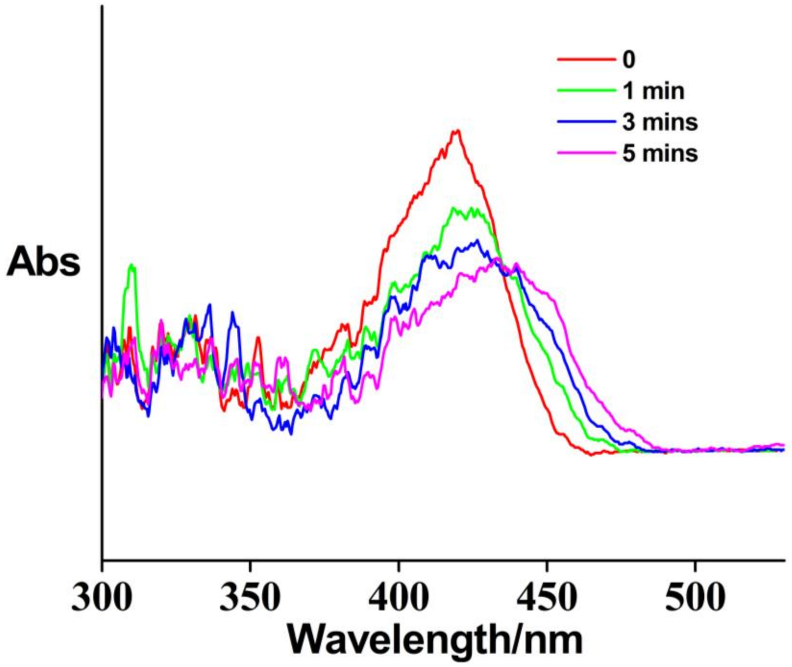

Supplementary Figure 21. Solid state absorption spectroscopy of photochromic process of TCA_NH_4@CHI_3 after 5 times irradiation and recovery.

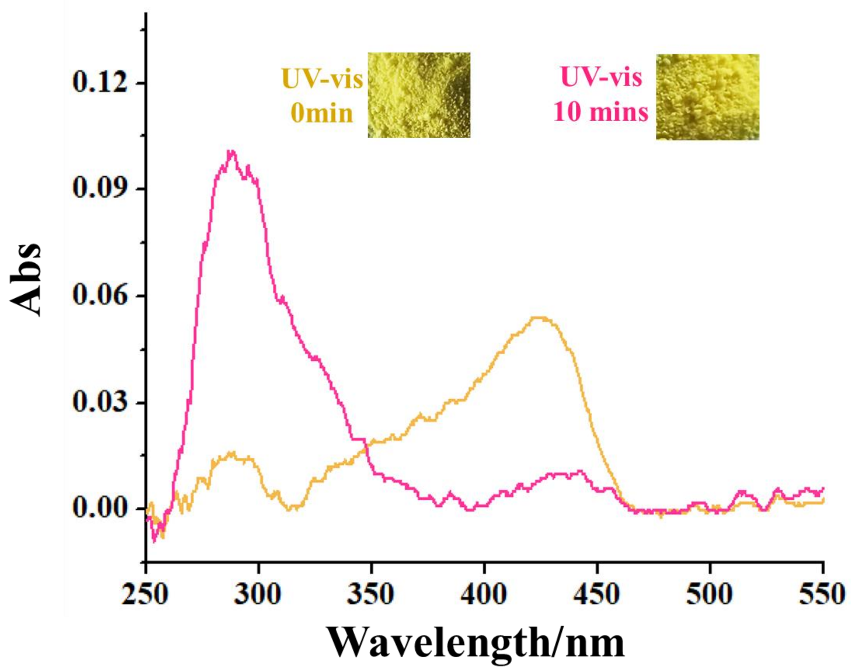

Supplementary Figure 22. Solid-state absorption spectrum of powder CHI_3 with UV-vis Xe lamp irradiation for 10 minutes.

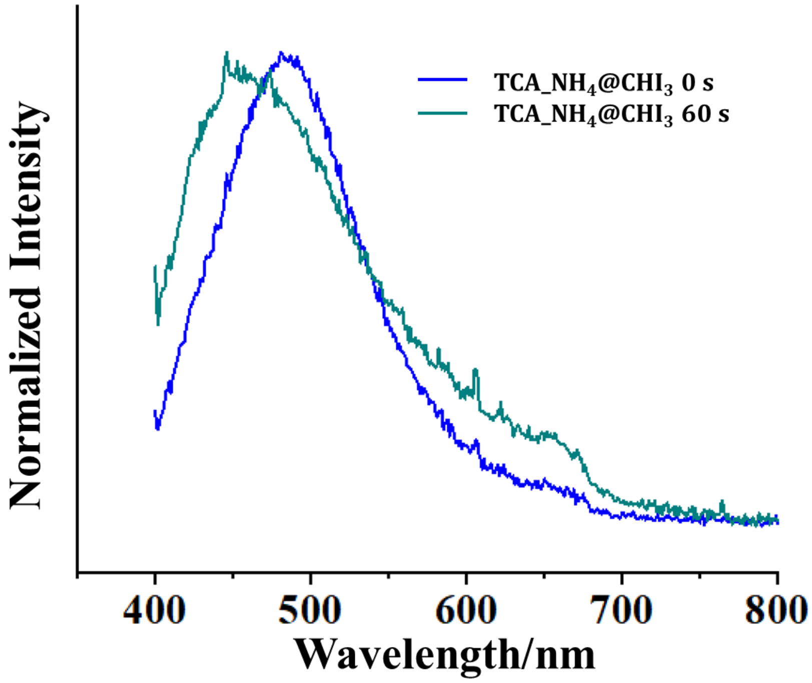

Supplementary Figure 23. The normalized emission spectrum of TCA_NH_4@CHI_3 before and after Xe lamp irradiation.

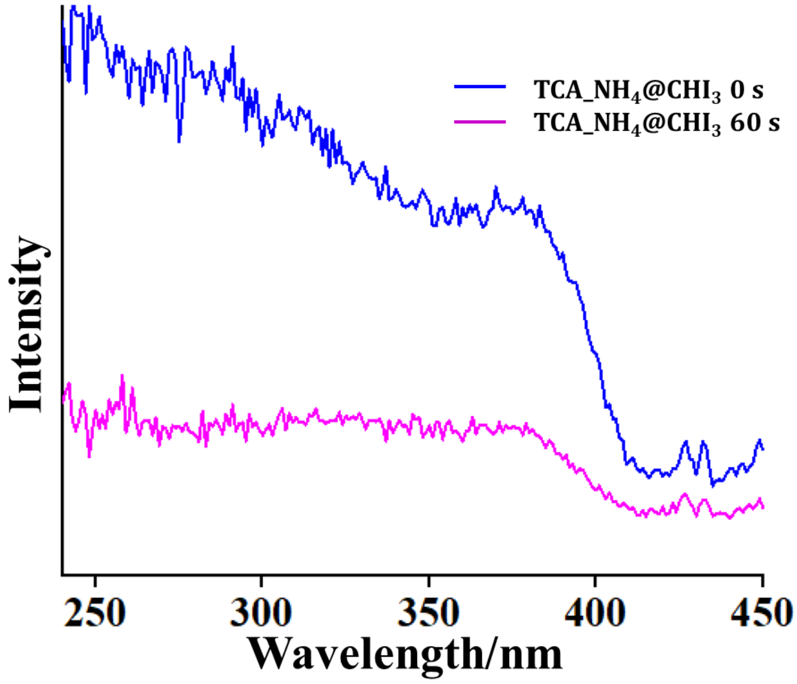

Supplementary Figure 24. The excitation spectrum of TCA_NH$_4$@CHI$_3$ before and after Xe lamp irradiation.
